# Data from the synthesis and characterization of banana starch nanoparticles from different botanical varieties

**DOI:** 10.1016/j.dib.2021.107167

**Published:** 2021-05-23

**Authors:** Natalia Baena-Jurado, Leidy T. Sanchez, Magda I. Pinzon, Cristian C. Villa

**Affiliations:** a Programa de Química, Facultad de Ciencias Básicas y Tecnologías, Universidad del Quindío. Carrera 15 Calle 12N, Armenia, Quindío, Colombia; b Programa de Ingeniería de Alimentos, Facultad de Ciencias Agroindustriales, Universidad del Quindío. Carrera 15 Calle 12N, Armenia, Quindío, Colombia

**Keywords:** Starch, Nanoparticles, Nanomaterials, Banana

## Abstract

In this work, we present data related to the structural features and thermal characteristics of starches from four varieties banana, likewise, we present the synthesis and characterization of starch nanoparticles from those starches. Data shows the structure of the starch granule and the nanoparticles are formed through XRD spectroscopy.

## Specifications Table

**Table:** 

Subject	Food Chemistry, Materials Sciences
Specific subject area	Starch based nanomaterials
Type of data	Tables and graphs.
How data were acquired	XRD, TGA, SEM
Data format	Analyzed
Parameters for data collection	Starch nanoparticles were synthetized from different banana species. They were characterized by different techniques
Description of data collection	Data was collected from several techniques, XRD, TGA and SEM
Data source location	Universidad del Quindío Armenia Colombia. Raw Data Format
Data accessibility	All raw data are available in this article and the following repository: https://data.mendeley.com/datasets/fpc6r9km5d/1

## Value of the Data

• Particle size and structural data of starch nanoparticles from different botanical banana varieties were obtained.
• Data can be used to improve starch nanoparticles synthesis methods.
• Properties of starch nanoparticles are dependent of their botanical source.

## 1 Data Description

### 1.1 XRD analyses

**Fig. 1:**
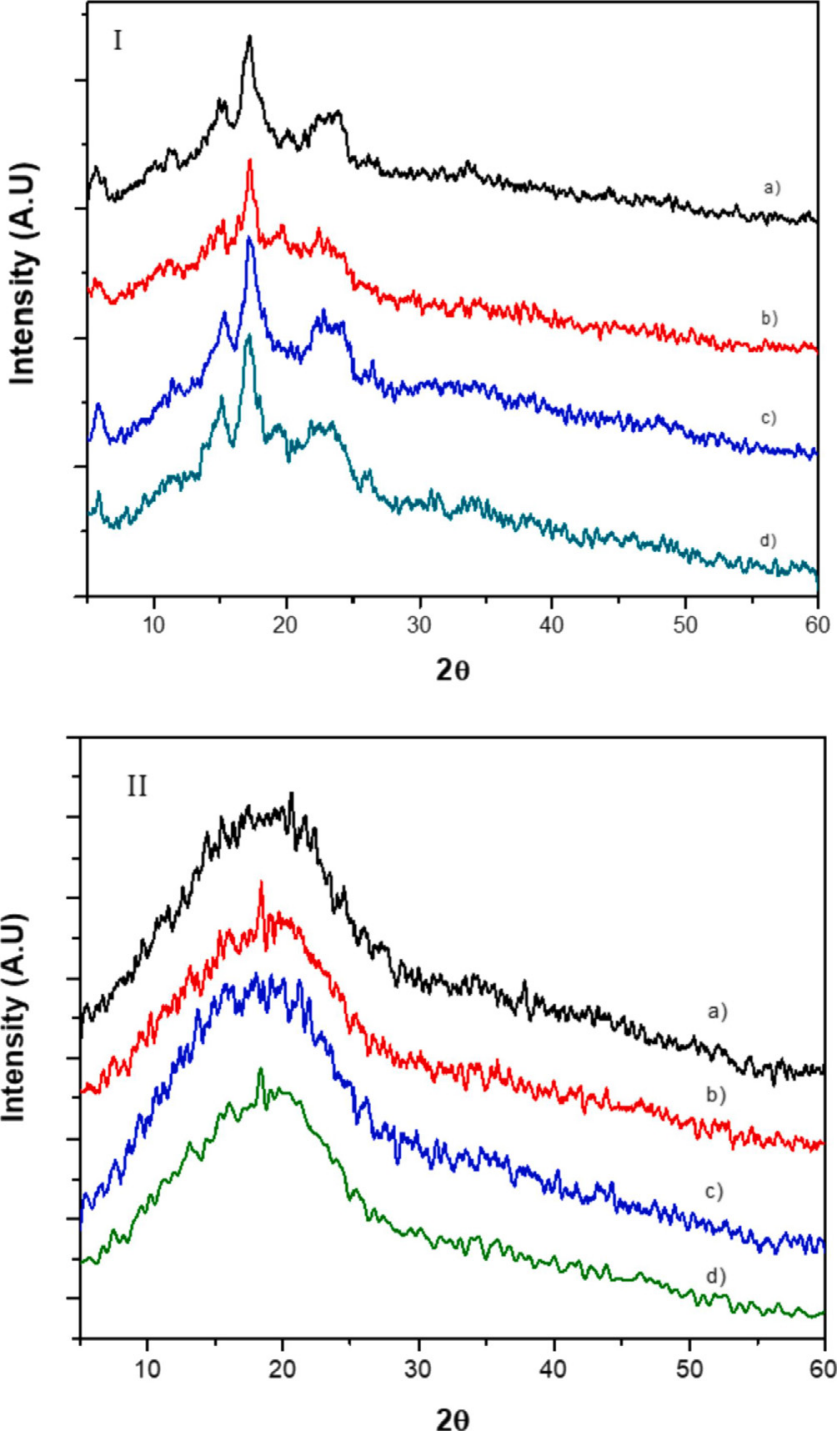
(I) XRD diffraction pattern of the different banana starches (a) S1; (b) S2; (c) S3; (d) S4 and (II) XRD diffraction patterns of the starch nanoparticles obtained from the different banana starches (a) SNP1; (b) SNP2; (c) SNP3; (d) SNP4.

Fig. 1(I) shows the XRD diffraction pattern of the different banana starch studied. All samples presented peaks at 2θ = 5.7°, 14.9°, 17.1°, 19.1°, 23.1° that correspond to the semi-crystalline nature of the granule and the C-type structure commonly assigned to starches. [1] Furthermore, Fig. 1(II) shows the XRD diffraction pattern of the different SNPs synthetized.

Table 1 shows the relative crystallinity (%Rc) of the different banana starches and SNPs studied. As shown in that table all four starches presented%Rc values in the range of 27–31%, those values are in accordance with previous reports for banana starches. [2], [3], [4] On the other hand, %Rc decreased considerably once the SNPs were formed, with values below 1%.

### 1.2 TGA analyses

**Fig. 2:**
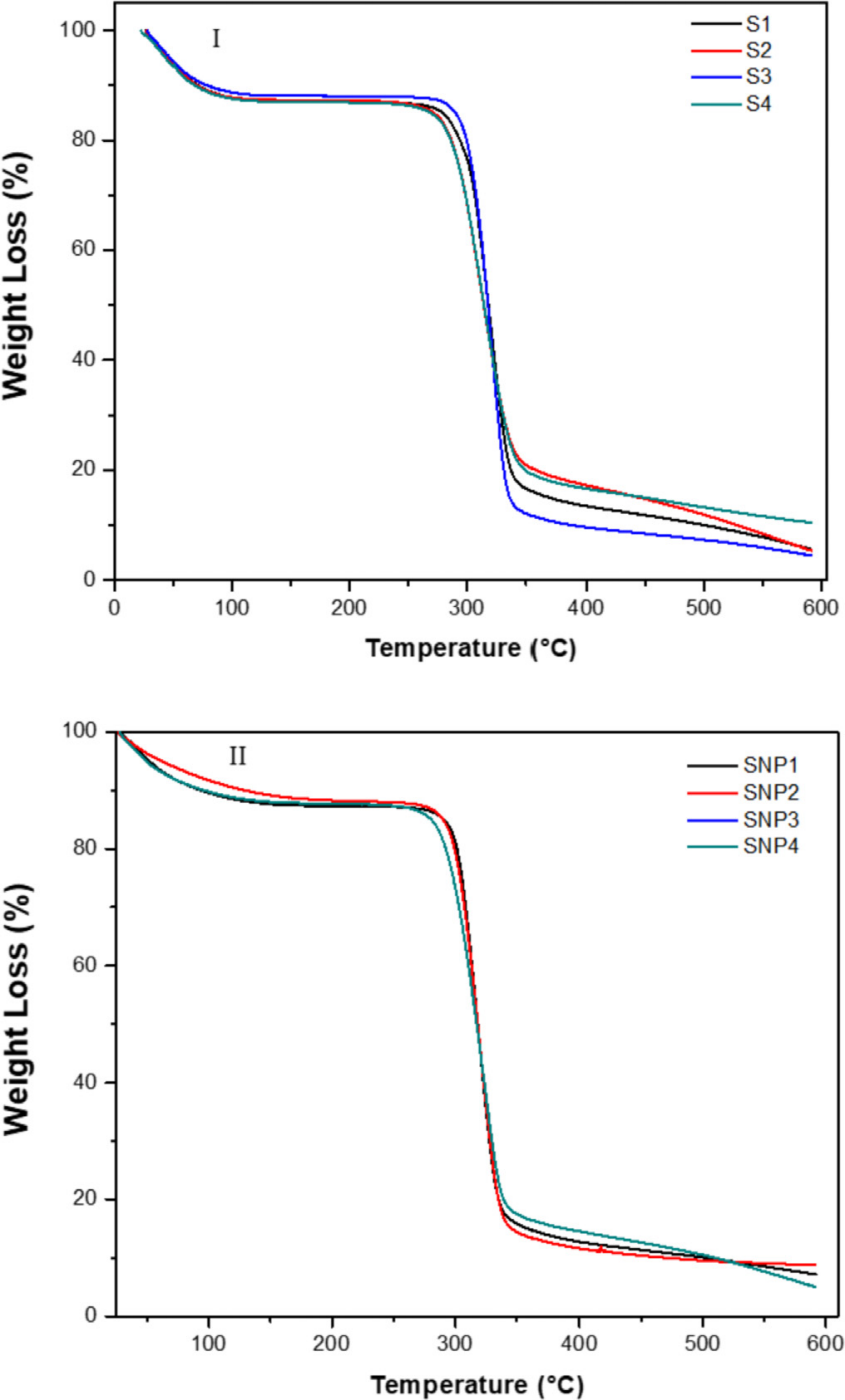
(I)  TG Curves of the different banana starch studies (II) TG curves of the different SNP studied.

**Fig. 3:**
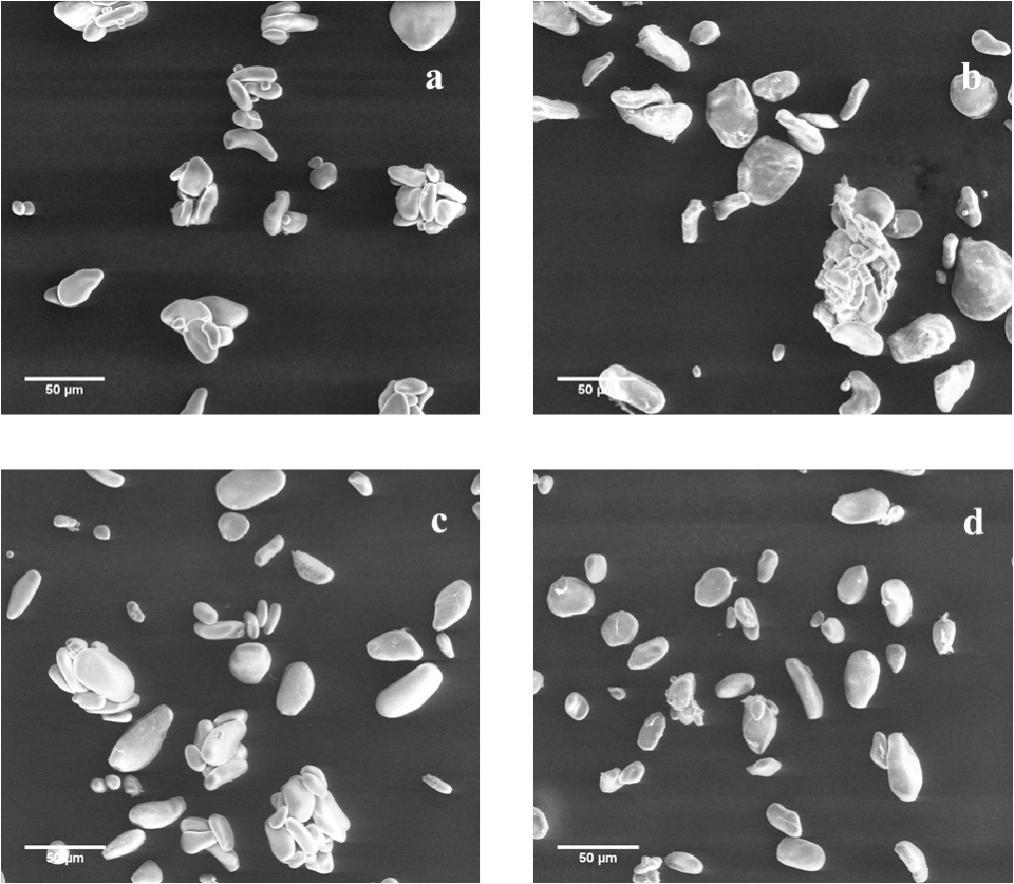
SEM images of the different banana starches (a) S1; (b) S2; (c) S3; (d) S4.

Fig. 2(I) shows the TG curves for the different banana starch studied. The first loss of mass corresponds to a percentage in the range 10–12% with no significant difference among them. A second mass loss beginning around 130–140 °C and ending around 350 – 360 °C. Finally, formation of ashes can be observed above 400 °C. Likewise, Fig. 3(II) shows the TG curves for the different SNP studied. The first mass loss corresponds 9–11%. There was not significant difference among the different SNPs studied.

**Table 1:** Relative crystallinity (%Rc) of the different banana starch and SNPs studied.

Starch	%Rc	SNPs	%Rc
S1	30.92 ± 2.12	SNP1	0.11 ± 0.06
S2	29.56 ± 1.31	SNP2	0.41 ± 0.12
S3	28.71 ± 1.43	SNP3	0.23 ± 0.09
S4	27.91 ± 3.11	SNP4	0.12 ± 0.07

### 1.3 SEM analyses

**Fig. 4:**
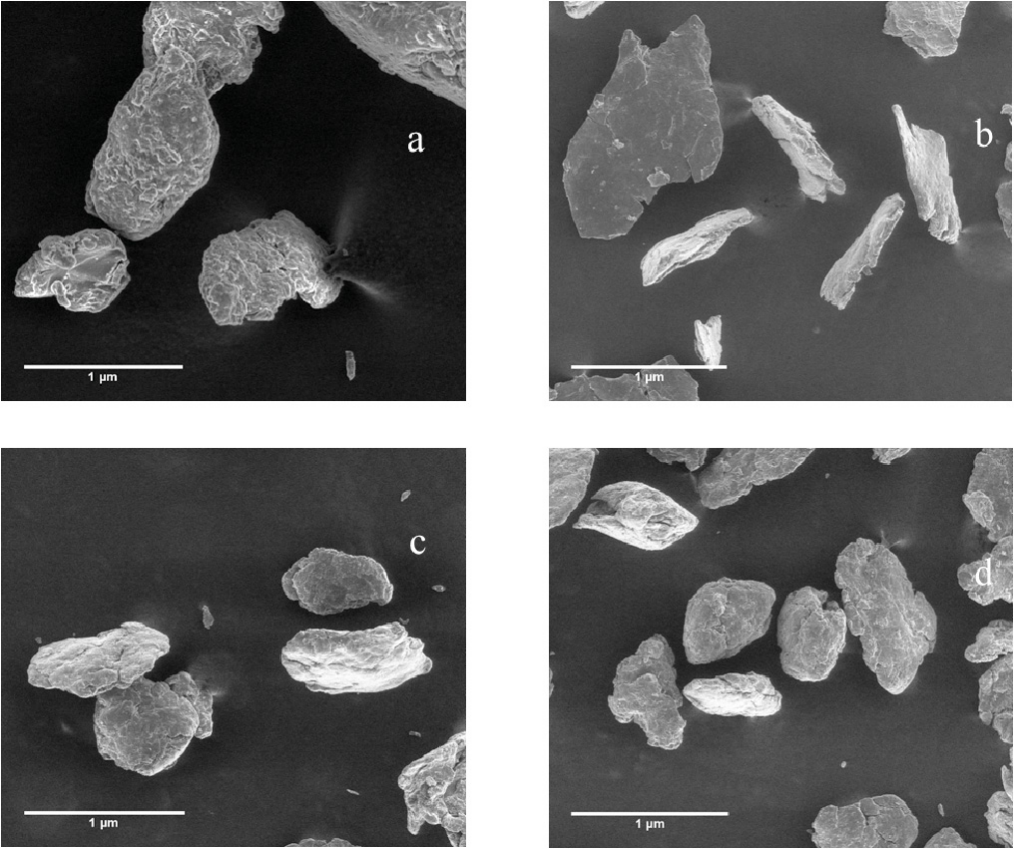
SEM images of the different banana starches (a) SNP1; (b) SNP2; (c) SNP3; (d) SNP4.

Starch granule size and morphology depend on the biochemistry of amyloplast, as well as physiology of the plant [5]. As shown in Fig. 3, the four starches presented two major shapes elongated oval and flattened spherical granules. Furthermore, starch granules presented a smooth and dense surface. Fig. 4 shows the SEM images of the different SNPs studied. All nanoparticles, presented and amorphous morphology with a rough surface, furthermore, particle sizes were in the range of 200–300 nm.

## 2 Experimental Design, Materials and Methods

### 2.1 Materials

Green bananas from all four varieties (Gros Michel, Cachaco, Manini and FHIA 21) where kindly provided by FEDEPLATANO from their crops in Quindío department in Colombia. These four varieties where chosen in order to have a representation of four different genomic groups. As a way to simplify reading, a codification of the four varieties was used: Gros Michel (S1), Cachaco (S2), Manini (S3), FHIA 21 (S4). All regents used in this studied were purchased from sigma (Analytical grade).

### 2.2 Starch isolation

Starch from the different banana varieties was isolated according to the procedure described in previous studies [18]. Peel was removed from banana samples and cut into slices. They were immediately dipped in a citric acid solution (2% w/v) for 5 min and then blended for 2 min. The suspension was then sieved, washed through screens several times and left to decant during 4 h. The starch sediments were dried in a Digitronic J.P Selecta hot air oven at 40 °C during 48 h.

### 2.3 Starch nanoparticles (SNPs) synthesis

Banana starch nanoparticles (SNPs) where synthetized using a nanoprecipitation method. [6]. In brief, 8 g of banana starch were mixed with 150 mL of destilled water, and heated at 80 °C with constant stirring (500 rpm) during 1 h. Once gelatinization occurred, the solution was left to cool to 25 °C, then 150 mL of ethanol where added dropwise (1 mL/min) with constant stirring (500 rpm). Finally, the solution was homogenized using an Ultraturrax at 10.000 rpm during 10 min, then the SNPs suspension was centrifuged at 4000 rpm during 30 min, and washed several times with ethanol to remove residual water. The SNPs were dried at 35 °C until all ethanol was removed.

### 2.4 X-Ray diffraction (XRD) spectroscopy

XRD spectroscopy analyses were carried out in a Bruker D8 Advance X-ray diffractometer operated at 40 kV and 100 mA with a Cu-K, radiation (λ= 1.54 Å. All samples were scanned through the 2θ range of 5 – 40°, with a continuous scan mode at room temperature. Relative crystallinity (%Rc) was calculated according to Eq. (1):

(1) $$\%Rc=\frac{A_{c}}{A_{c}+A_{a}}\times 100$$

Where Ac is the area of the crystalline region and Aa is the area of the amorphous region of the diffraction pattern.

### 2.5 Thermogavimetric analyses (TGA)

Thermogavimetric (TGA) were carried in a Q500 TGA TA Instruments equipment. Samples (2–5 mg) were heated from 25 °C to 600 °C at a 10 °C/min rate under a N_2_ atmosphere (20 mL/min).

### 2.6 Electron scanning microscopy (SEM)

Scanning Electron Microscopy (SEM) analyses were carried out in a FEI QUANTA 250 SEM microscope (Oberkochen, Germany). Samples were placed on circular aluminum stubs with double-sided carbon adhesive tape and images were captured at different magnifications.

## CRediT Author Statement

**Natalia Baena-Jurado:** Methodology, Investigation; **Leidy T. Sanchez:** Data curation, Writing - Original draft preparation; **Magda I Pinzon:** Data curation, Writing - Original draft preparation; **Cristian C. Villa:** Writing - Reviewing and Editing.

## Supporting Information

Zip File containing SEM images, TGA and XRD data.

## Ethics Statement

The research does not involve human subjects, animal experiments or data collected from social media platforms.

## Declaration of Competing Interest

The authors declare that they have no known competing financial interests or personal relationships which have or could be perceived to have influenced the work reported in this article.

## Appendix Supplementary materials

Image, application 1

Image, application 2
