# Supplementary figures and images for: Data from the synthesis and characterization of banana starch nanoparticles from different botanical varieties
Source: Data Brief. 2021 May 23;37:107167. doi: 10.1016/j.dib.2021.107167 (PMC8170071; doi:10.1016/j.dib.2021.107167)

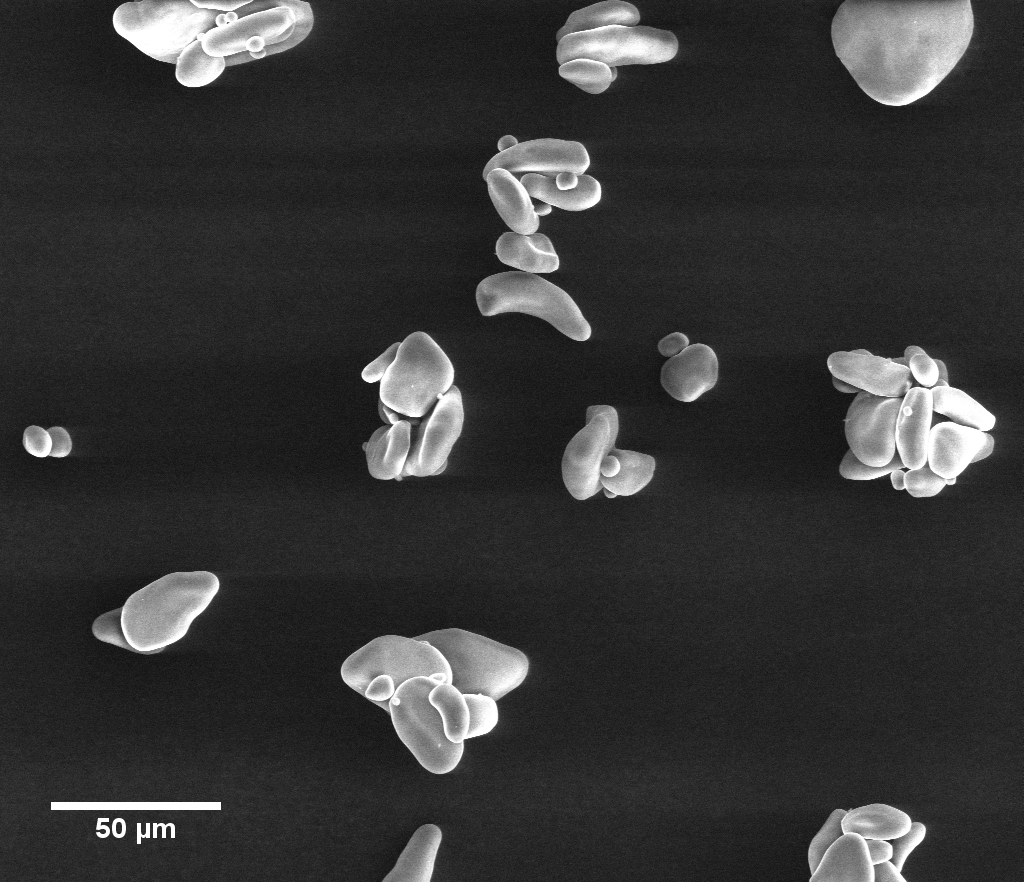

Supplement: Supplementary file 2 [file mmc2.zip › Starch A1.tif]

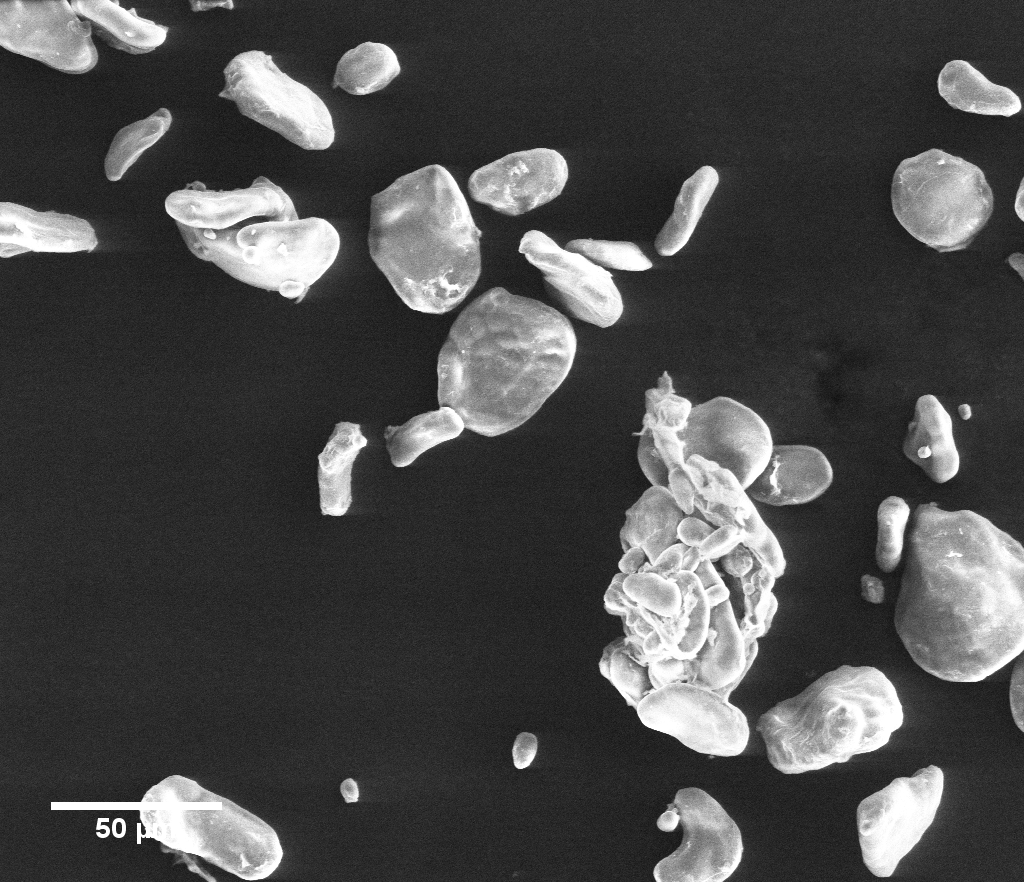

Supplement: Supplementary file 2 [file mmc2.zip › Starch A2.tif]

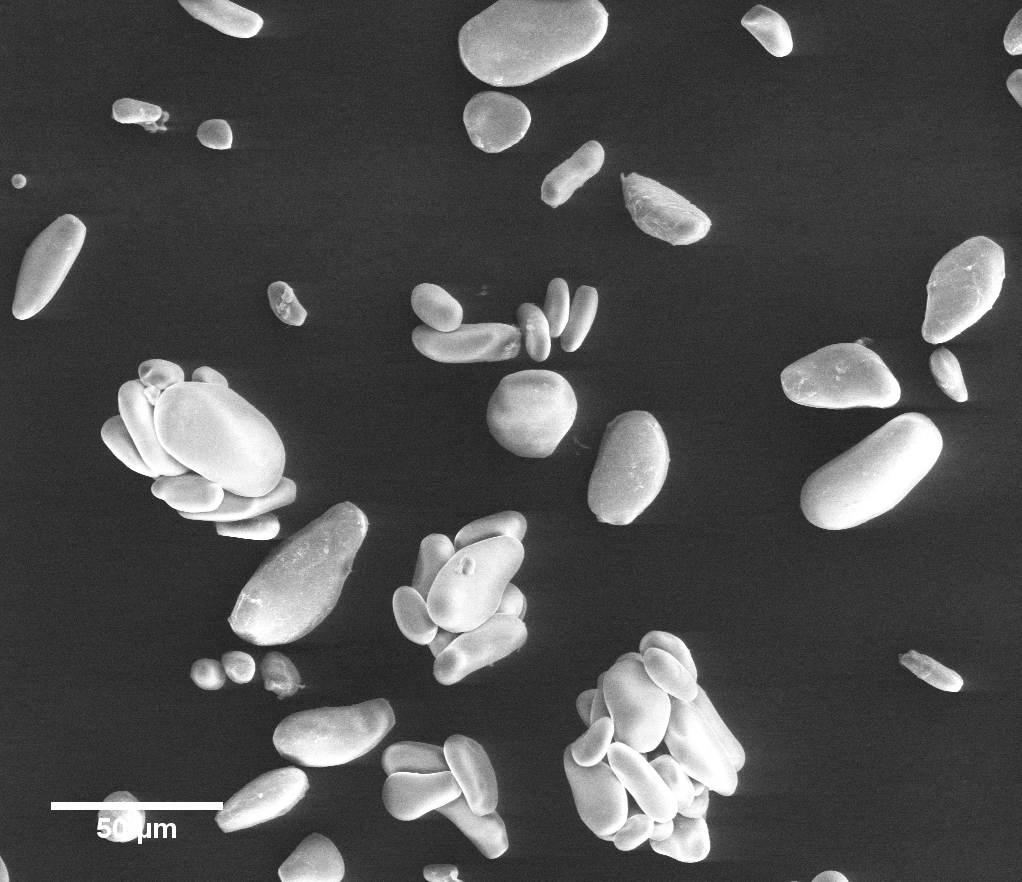

Supplement: Supplementary file 2 [file mmc2.zip › Starch A3.tif]

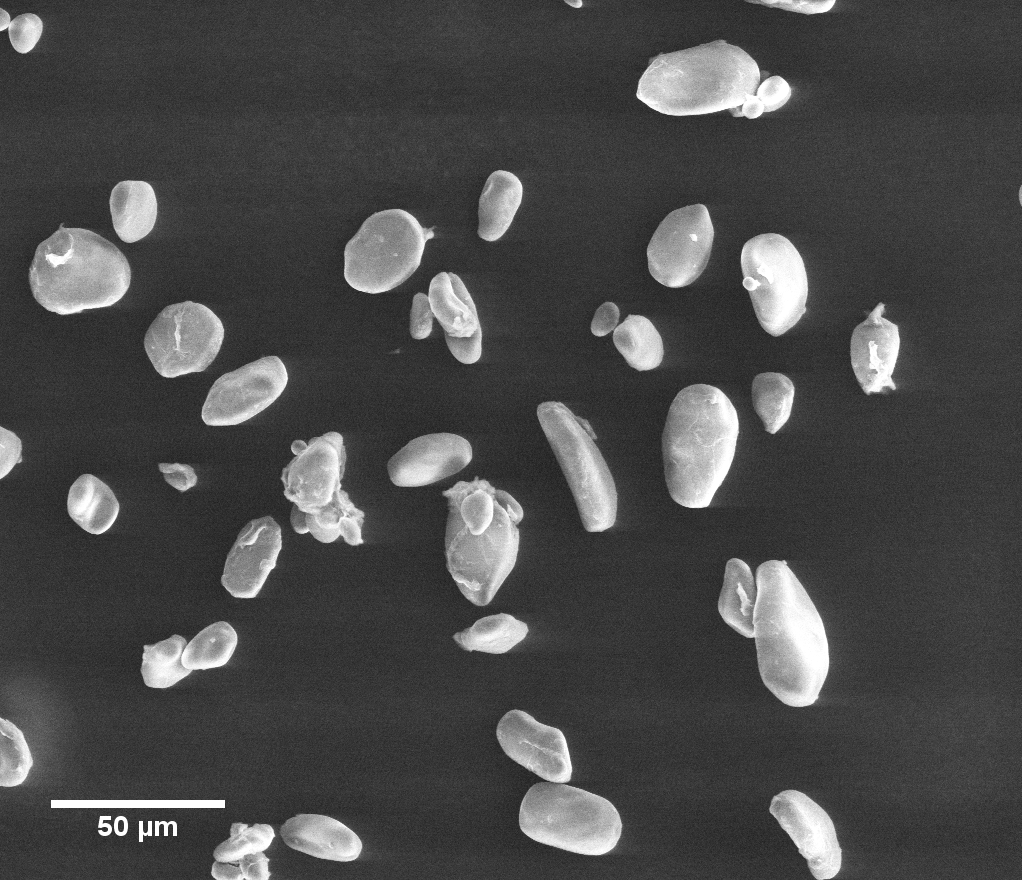

Supplement: Supplementary file 2 [file mmc2.zip › Starch A4.tif]
